# Engaging stakeholders in the use of an interactive simulation tool to support decision-making about the implementation of colorectal cancer screening interventions

**DOI:** 10.1007/s10552-023-01692-0

**Published:** 2023-05-06

**Authors:** Meghan C. O’Leary, Kristen Hassmiller Lich, Maria E. Mayorga, Karen Hicklin, Melinda M. Davis, Alison T. Brenner, Daniel S. Reuland, Sarah A. Birken, Stephanie B. Wheeler

**Affiliations:** 1 Department of Health Policy and Management, Gillings School of Global Public Health, University of North Carolina at Chapel Hill, Chapel Hill, NC USA; 2 Lineberger Comprehensive Cancer Center, University of North Carolina at Chapel Hill, Chapel Hill, NC USA; 3 Department of Industrial and Systems Engineering, North Carolina State University, Raleigh, NC USA; 4 Department of Industrial and Systems Engineering, Herbert Wertheim College of Engineering, University of Florida, Gainesville, FL USA; 5 Oregon Rural Practice-Based Research Network, Oregon Health & Science University, Portland, OR USA; 6 Department of Family Medicine, Oregon Health & Science University, Portland, OR USA; 7 School of Public Health, Oregon Health & Science University, Portland State University, Portland, OR USA; 8 Division of General Medicine and Clinical Epidemiology, Department of Medicine, University of North Carolina School of Medicine, Chapel Hill, NC USA; 9 Center for Health Promotion and Disease Prevention, University of North Carolina at Chapel Hill, Chapel Hill, NC USA; 10 Department of Implementation Science, Wake Forest School of Medicine, Winston-Salem, NC USA

**Keywords:** Decision-making, Simulation, Implementation, Evidence-based intervention, Colorectal cancer screening

## Abstract

**Purpose:**

We aimed to understand how an interactive, web-based simulation tool can be optimized to support decision-making about the implementation of evidence-based interventions (EBIs) for improving colorectal cancer (CRC) screening.

**Methods:**

Interviews were conducted with decision-makers, including health administrators, advocates, and researchers, with a strong foundation in CRC prevention. Following a demonstration of the microsimulation modeling tool, participants reflected on the tool’s potential impact for informing the selection and implementation of strategies for improving CRC screening and outcomes. The interviews assessed participants’ preferences regarding the tool’s design and content, comprehension of the model results, and recommendations for improving the tool.

**Results:**

Seventeen decision-makers completed interviews. Themes regarding the tool’s utility included building a case for EBI implementation, selecting EBIs to adopt, setting implementation goals, and understanding the evidence base. Reported barriers to guiding EBI implementation included the tool being too research-focused, contextual differences between the simulated and local contexts, and lack of specificity regarding the design of simulated EBIs. Recommendations to address these challenges included making the data more actionable, allowing users to enter their own model inputs, and providing a how-to guide for implementing the simulated EBIs.

**Conclusion:**

Diverse decision-makers found the simulation tool to be most useful for supporting early implementation phases, especially deciding *which* EBI(s) to implement. To increase the tool’s utility, providing detailed guidance on *how* to implement the selected EBIs, and the extent to which users can expect similar CRC screening gains in their contexts, should be prioritized.

**Supplementary Information:**

The online version contains supplementary material available at 10.1007/s10552-023-01692-0.

## Background

Health organizations face many decisions related to the selection and implementation of interventions intended to address existing gaps in colorectal cancer (CRC) screening. Multiple types of evidence-based interventions (EBIs), such as stool testing-based outreach, patient navigation, and patient reminders, have been shown to improve CRC screening in diverse populations and settings [1–5]. However, there are challenges related to selecting one or more EBIs that best fit the local context, assessing organizational readiness for EBI implementation, and effectively planning for the resources required for EBI implementation [3, 6]. Clinic administrators have previously reported little systematic selection or evaluation of interventions [7]. Prior research has also shown that the degree of EBI implementation is associated with CRC screening outcomes [8]. Low implementation due to challenges such as competing clinic demands and staff turnover can threaten the effective coverage and sustainability of EBIs [9–11]. Given the large investment of resources required to implement EBIs, regardless of their outcomes, it is critical for organizations to select and adopt EBIs that are most appropriate for their context.

Simulation modeling has been used to estimate and forecast the financial and health impact of various interventions intended to improve CRC screening delivery and associated outcomes such as CRC incidence and mortality [12–14]. These models generally aim to provide insight into the changes expected in the future under different decision alternatives in order to facilitate evidence-informed decision-making [13, 15]. For example, these models can provide guidance regarding which EBIs may be most cost-effective in the long-term, support the largest gains in the prevalence of CRC screening in diverse populations, and be most feasible to implement in terms of initial budgets and existing infrastructure. In fact, modeling and simulating the proposed EBIs or other types of changes prior to implementation has been recognized as an implementation strategy (i.e., a method used to support and strengthen EBI adoption, implementation, and sustainment) by experts in implementation research [16].

However, while simulation models have the potential to address the types of challenging questions faced by decision-makers about EBI implementation, it remains unclear the extent to which these models are being used by their target end users. Smith et al., for example, identified a total of 43 studies published from 2008 to 2019 that described simulation models focused on improving CRC screening delivery [12, 13]. Although 91% of the models included in their systematic review were designed with the intention of informing health or policy-relevant decision-making, they found that just 12% of included studies reported an impact on data-informed decision-making (although separate correspondence with some model developers indicated that additional models informed decisions that were not reported) [12]. Furthermore, more information is needed on how decision-makers would ideally prefer to engage with these models when considering EBI implementation decisions.

In this study, we aimed to pilot an interactive, web-based simulation modeling tool that presents data on the projected health and financial impact of EBIs on CRC screening and outcomes. Our primary objective was to understand how the simulation tool could be used to guide decision-makers in selecting and implementing CRC screening EBIs. We also aimed to understand how to optimize the utility of this type of tool for different types of decision-makers by assessing participants’ preferences about and comprehension of the interactive tool features and simulation results.

## Methods

### Overview

This study used interviews with individuals involved in making decisions about CRC prevention and control efforts to assess how the simulation modeling tool could be used to inform decisions about the selection and implementation of EBIs intended to improve CRC screening. Below, we describe the simulation modeling studies that informed this work, the development of the simulation tool, the types of decision-makers recruited, and the structure and analysis of the interviews. This study was exempt from review by the Institutional Review Board at the University of North Carolina at Chapel Hill.

### Population simulation

Simulation modeling uses existing data to create a virtual representation of reality in order to forecast future outcomes associated with various decision alternatives. Our team previously conducted a series of microsimulation studies in order to compare the effectiveness and cost-effectiveness of different EBIs across a range of short-term and long-term outcomes, such as the percentage of eligible adults screened for CRC, number of CRC cases averted, implementation, screening, and treatment costs, and cost per additional person-year up-to-date on CRC screening [17–22]. Microsimulation is a modeling approach in which individuals with diverse characteristics can transition between health states and have their outcomes tracked over time [23–25]. By tracking their individual outcomes in response to multiple alternatives, we can assess the population-level impact of different potential interventions on pre-specified outcomes. In our case, we evaluated and reported on the expected changes in CRC screening and related outcomes associated with EBIs and policy changes compared to usual care in two contexts—the Oregon Medicaid population [17] and the North Carolina state population [18–20].

### Interactive simulation tool

The goal of our microsimulation studies was to inform more evidence-based decision-making [15] about how to equitably and efficiently improve CRC screening and downstream CRC outcomes (e.g., incidence, mortality) among age-eligible, average-risk adults through the implementation of individual or multicomponent EBIs. To make the results of these simulation studies more accessible to decision-makers, we developed a web-based simulation tool called Cancer Control PopSim (Population Simulation), available at https://popsim.org/. The Cancer Control PopSim tool, as well as the research studies reported on this tool, were all supported by the Cancer Prevention and Control Research Network (CPCRN). The tool was intended to inform decision-making in two ways. First, it uses visualization and corresponding text to aid users in reviewing and understanding our simulation results, given the specific modeling assumptions we made about the effectiveness, reach, and cost of the simulated EBIs, in an interactive format. Second, it allows users to conduct their own sensitivity analyses [26] where they can vary some of our modeling assumptions to better reflect their own contexts. For example, users have the opportunity to scale up or down the assumed implementation costs, depending on their existing infrastructure and resources needed, and determine how these changes would affect the model results.

**Fig. 1:**
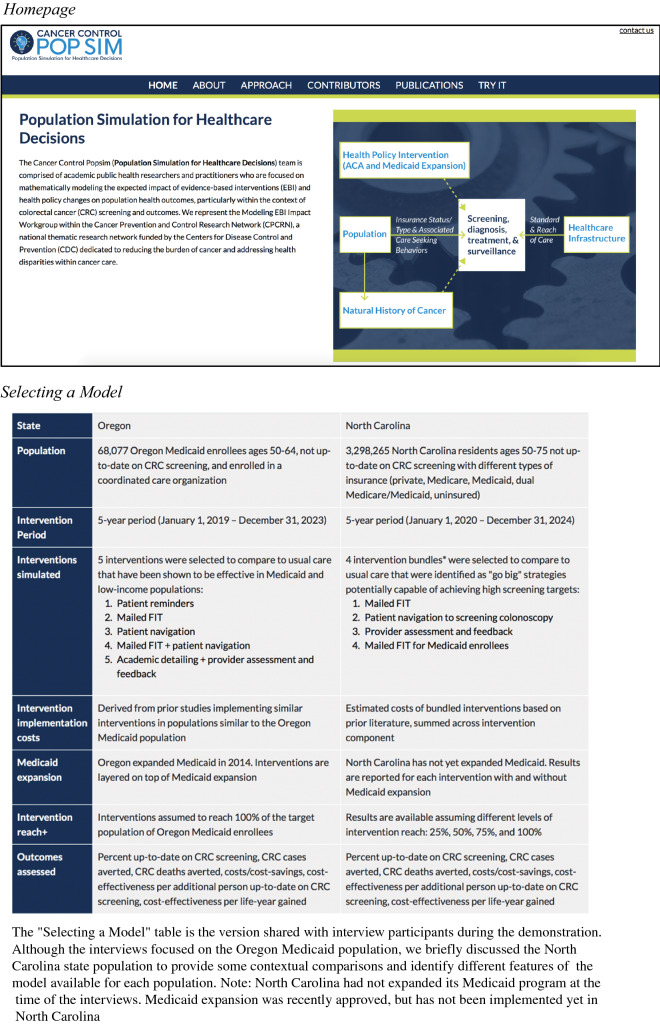
Snapshot of the Cancer Control PopSim tool

The Cancer Control PopSim tool uses a step-by-step process to guide users through the following steps: (1) review the evidence base for the simulated EBIs, (2) select the specific EBIs to compare to usual care in terms of their screening outcomes, long-term health impact, and costs, (3) understand and adjust as needed our modeling assumptions, (4) review the simulated outcomes overall and by specific subgroups (e.g., by race, ethnicity, age, geographic location, insurance type, stage of cancer diagnosis), and (5) assess the cost-effectiveness of the EBIs compared to usual care. Figure 1 provides a snapshot of the tool and a summary of the available models. At the time this study was conducted, the tool was not yet publicly available. Instead, we presented a prototype of the tool that included a limited number of options to focus on how this type of tool could be used to guide decision-making about strategies for improving CRC screening. For example, we presented only the Oregon Medicaid model and showcased a subset of the simulated EBIs and modification options, thus ensuring that users considered the types of features available and how this tool could be utilized in general, rather than evaluating and interpreting the simulation results themselves.

### Participants and recruitment

Individuals were eligible to participate in this study if they performed one or more decision-making roles related to CRC screening in their current professional roles. Participants did not need to have any prior experience conducting or using simulation modeling. Instead, we prioritized recruiting a diverse mix of decision-makers who each had a strong foundation in CRC prevention and control efforts and, thus, could provide insight into how to optimize the simulation tool to inform the implementation of EBIs to support CRC screening. Since few participants reported any prior experience with simulation modeling, they were also able to share feedback about how easy or difficult they found it to understand the simulation tool, methods used, and types of data presented. Additionally, they provided suggestions for improving comprehension of the tool to better support potential users who are invested in improving CRC screening and outcomes but are new to these types of methods.

We used a purposive sampling approach to recruitment to ensure that interview participants represented a wide range of decision-making roles, types of organizations involved in CRC prevention and control, and years of experience. For example, we aimed to reach decision-makers working in smaller and more resource-constrained healthcare settings, larger and more integrated healthcare settings, government agencies that fund CRC screening initiatives, non-government organizations that advocate for CRC screening programs or inform screening recommendations, and academic institutions that partner with health organizations on EBI implementation. Since the Cancer Control PopSim tool currently includes North Carolina- and Oregon-specific models, we prioritized reaching those who serve the southeastern or northwestern regions of the US and, therefore, have a good understanding of these respective populations. We also recruited those whose decision-making roles influence CRC screening programs at the national level to understand the extent to which potential users outside of these two specific regions perceive being able to use the tool.

We developed an initial list of possible participants to recruit by leveraging our existing cancer prevention and control networks. This included identifying decision-makers using attendee lists from state-level and national-level meetings focused on CRC screening and obtaining recommendations from experts in this field. During the interviews, we also asked participants if they had any recommendations for decision-makers we should invite to participate. Recruitment occurred via email, with up to two reminder emails sent as needed. We conducted interviews until saturation of themes was reached, and we had a representative mix of different types of decision-makers. Participants received a $50 gift card for completing the interview.

### Data collection

Interviews were conducted virtually, lasting up to one hour, and consisted of two parts: (1) demonstration of the simulation tool using a standardized script and (2) semi-structured interview to assess participants’ comprehension of the tool, preferences related to the content and design of the tool, and perceptions of the utility of the tool in informing EBI selection and implementation. The demonstration took approximately 6–8 min and provided an overview of the EBIs and populations simulated, the step-by-step process used to guide users through interacting with the tool, and brief examples of the types of simulation results reported and how to interpret those results. We piloted the demonstration and accompanying script with two doctoral students trained in simulation modeling but not involved in this project. The script was updated to reflect their feedback, such as building in short definitions of simulation modeling, life-years, and other research terms for a lay audience, and providing specific examples of how to interpret the assumed effectiveness and cost of simulated EBIs. During the demonstration, participants had the opportunity to ask any clarifying questions about the tool content, which aided in identifying possible areas of confusion.

In the interview guide, we included a short section intended to assess participants’ initial reactions to the tool having just viewed the demonstration. For each of six different statements, participants were asked to rank their agreement using a 5-point Likert scale (i.e., strongly disagree to strongly agree). These initial reactions provided opportunities to probe on ways to increase comprehension and the utility of the tool among diverse types of users. The interview guide also included open-ended questions intended to assess participants’ perceptions of the benefits and challenges of using this tool as an implementation strategy to inform decision-making, which individuals and organizations should be the target users of this tool, and how to improve the visualization and accompanying text for optimal use by those audiences. Examples of questions included the following: considering your other strategies for informing efforts to improve CRC screening, how might you use this tool in your decision-making role; what might prevent you from using this tool to support decision-making; and what would help to make it easier to understand or interpret [the model results]. The Supplement includes the complete demonstration script and interview guide.

### Analysis

All interviews were audio-recorded and transcribed verbatim. For each of the statements assessing participants’ immediate reactions to the tool using a Likert scale, we determined the mean, median, minimum, and maximum values across participants. We then coded the qualitative text using template analysis [27]. Since our primary goal was to optimize the simulation tool to meet the needs and interests of diverse users, we created codes related to the types of intended users, strengths of the tool, and challenges of the tool based on participants’ responses, focusing specifically on decision-making about EBI implementation. We tracked any recommendations for improving the overall utility, design, and content of the tool. We also identified exemplary quotes illustrating each theme, and reported the frequency with which each theme was reported in the tables.

## Results

Seventeen decision-makers participated in interviews about the Cancer Control PopSim tool between June and August 2022. Most participants completed individual interviews, although one group interview including three participants was conducted for logistical reasons. Table 1 presents the participants’ characteristics. Briefly, the participants represented a mix of decision-making roles related to CRC prevention and control, including researchers who engage with and train health partners on CRC screening efforts (35%), clinic administrators/staff (29%), government employees who provide funding, training, and technical assistance to health organizations (29%), and non-governmental organization (NGO) employees and advocates (18%). There was a nearly equal number of participants who have served in their current decision-making roles for less than 5 years (47%) versus 5 years or longer (53%), with approximately one-third (35%) of all participants reporting 10 years or more of experience in their current roles.

**Table 1:** Characteristics of participants interviewed about the simulation tool

Participant characteristic	*n* (%)
Overall	17 (100)
Sex	
Female	14 (82)
Male	3 (18)
Region served*	
Federal	7 (41)
Northwest	7 (41)
Southeast	7 (41)
Type of decision-maker*	
Clinic administrator/staff	5 (29)
Government employee	5 (29)
Non-government/advocacy organization employee	3 (18)
Researcher**	6 (35)
Number of years in current role^+^	
Less than 2 years	4 (24)
2 years to less than 5 years	4 (24)
5 years to less than 10 years	3 (18)
10 years or more	6 (35)

*These categories are not mutually exclusive. Participants may serve multiple regions through their roles if, for example, they are involved in the implementation of EBIs in their local communities and also provide input or recommendations about EBI implementation to healthcare organizations and other entities nationally. In addition, some participants reported performing multiple decision-making roles, for example, as both a clinic administrator/staff and researcher

**Researchers interviewed are considered decision-makers because they inform and/or support implementation of CRC screening EBIs in other settings, including but not limited to clinics, health departments, and pharmacies

^+ ^Some participants reported additional years of expertise with CRC prevention and control outside of their current role

### Intended users

Participants identified a combined list of 30 possible end users for the simulation tool. The list included broad categories of organizations, such as health systems, payers, advocacy groups, and population health programs, and specific organizations like the National Colorectal Cancer Roundtable and the National Cancer Institute. However, participants disagreed on whether dissemination of the tool should prioritize health organizations of different sizes or only those that are larger and have more widespread influence. For example, a researcher serving the northwest region commented, “[Intended users] are kind of opposite spectrums. Like smaller, more resource-constrained organizations would really benefit, but also the very large organizations, health plans, and health systems could really benefit from this tool.” In contrast, a clinician/researcher serving the federal and northwest regions recommended prioritizing dissemination at the higher level: “If you can influence the public health system to do this, or the state health authority, then you don’t have to worry about the clinics, because they’re paying for it. They say [clinics] have to do it.” Participants’ perceptions of the target users shaped their feedback on the ways in which the tool can be used to inform EBI implementation.

### Initial reactions

Table 2 reports participants’ level of agreement with six summary statements about the tool using a 5-point scale and assessed immediately after they viewed the tool demonstration. Participants agreed, on average, with three of these statements: (1) this tool could help to implement strategies for improving CRC screening more effectively (mean: 4.4, median: 5.0); (2) they would like to use tools like this to support decision-making about strategies for improving CRC screening frequently (mean: 4.4, median: 4.0); and (3) the various tool components were well-integrated (mean: 4.3; median: 4.0). Participants were generally neutral on whether the tool makes them think differently about strategies for improving CRC screening (mean: 3.4, median: 3.0), and whether most people in healthcare could learn to use this tool quickly (mean: 3.4; median: 4.0). While neutral, on average, participants differed on whether they would need to learn more about simulation modeling to be able to use this tool, with their responses ranging from strongly disagree to strongly agree (mean: 2.9; median: 3.0). Participants' questions immediately following the tool demonstration included clarifications on how to interpret EBI cost-effectiveness and details about the particular elements included in the microsimulation model.

**Table 2:** Participants’ immediate reactions about the interactive simulation tool following tool demonstration (*n* = 17)

Statement about the simulation tool	Mean	Median	Range^a^
I think that I would like to use tools like this to support decision-making about strategies for improving CRC screening frequently	4.4	4.0	4–5
This tool makes me think differently about strategies for improving CRC screening	3.4	3.0	2–5
I found the various components of this tool were well-integrated	4.3	4.0	3–5
I think that I would need to learn more about simulation modeling to be able to use this tool	2.9	3.0	1–5
I would imagine that most people in healthcare could learn to use this tool quickly	3.4	4.0	2–5
I think this tool could help to implement strategies for improving CRC screening more effectively	4.4	5.0	3–5

These statements were assessed immediately following the standardized tool demonstration that took place at the start of each interview, before participants had an opportunity to explore the tool independently

^a^Statements were evaluated on a 5-point Likert scale: strongly disagree (1), disagree (2), neither agree nor disagree (3), agree (4), strongly agree (5)

### Utility of simulation tool for informing EBI implementation

Table 3 summarizes the key themes related to decision-makers’ perceptions of the utility of the Cancer Control PopSim tool for informing EBI implementation, including the frequency with which each theme was reported and an illustrative quote describing the theme. Benefit of this tool to be the ability to use model inputs and outputs to build a case supporting EBI adoption and implementation when seeking buy-in from higher-level decision-makers, including senior leadership and policymakers. Participants also discussed the utility of this tool with respect to selecting which EBI or combination of EBIs to implement in different settings based on the simulated data.

**Table 3:** Themes regarding the utility of the simulation tool for informing EBI implementation (*n* = 17)

Theme	Theme description	# (%) Respondents	Exemplary quote
Building a case	Using the model inputs and outputs to clearly demonstrate to diverse decision-makers (e.g., senior leadership, policymakers) the rationale for EBI implementation	12 (71%)	“And the further removed you are from the clinic…the way we have to communicate to each other changes….I can tell a heartwarming story about how I had to navigate a patient and got them linked to care, but I can also follow up with data and projections and sustainability of plans that are going to speak volumes to senior leadership.”
Selecting EBIs	Selecting which EBI(s) to implement in the local setting through a data-driven approach	11 (65%)	“It facilitates using data for decision making…we want the very data informed decisions about what to implement.”
Setting goals, prioritizing	Identifying priorities (e.g., addressing screening disparities, reaching quality metrics, selecting cost-effective EBIs, etc.) upfront and using the tool to determine how to achieve those goals	6 (35%)	“You can balance your approach..in a way that you can have a portfolio of pilots that are going to be a good return on investment plus impact across folks who need access.”
Acting as a literature review	Using this tool as a type of literature review to understand the current evidence base or as a way to inform grant and proposal writing for CRC screening programs	6 (35%)	“This could be a very handy tool to start with to see what are some models that are out there right now, what are the inputs that they’re using, what’s the evidence base for using those inputs.”
Facilitating discussion with health partners	Engaging clinics and other health partners in discussions around their options for EBI implementation and using this tool to provide education or to address their questions	5 (29%)	“I think we would definitely use this when we’re having initial conversations with partners. It always comes up. What’s the cost or what kind of cost can we expect? I love that you guys have training costs in there as well, because we speak about that very vaguely, or we’ll say…we had clinics that did it this way and it was very minimal, or there was a centralized person, or you can use a front desk person to do this, not an RN…so trying to really outline those strategies. Your tool provides an excellent roadmap to have those conversations.”
Reinforcing decisions or recommendations about EBI selection	Using the tool to support or strengthen existing recommendations about which EBI(s) to implement	3 (18%)	“It’s nice because this kind of supports my existing opinion about what…should we be doing and what’s effective and…what should we be pushing for, so I mean I would use this if I were going to make the argument for what…should we be doing more of. I would use this to support that.”
Increasing prioritization of CRC screening	Providing evidence for why prioritizing CRC screening initiatives is important given other competing population-level health problems	3 (18%)	“…colorectal [cancer screening] is not a huge priority on people’s plate these days. Opiates for the FQHCs [federally-qualified health centers] is bigger and obviously COVID. So you have to come in there and say this is really going to…make things better for clinicians, and it’s cost saving…if you can take this off their plate you’re going to get higher screening rates and it’s going to be low cost compared to all these other things you have to do. Because otherwise they’re not going to listen, because there are too many other higher priorities.”

Additional opportunities for the simulation tool to inform decision-making included the setting and prioritization of goals, and using the tool as a literature review to understand the evidence base. With respect to goal-setting, decision-makers discussed their individual or respective organization’s priorities for improving CRC screening and outcomes and how the tool could facilitate identifying opportunities to best balance those priorities. This included, for example, selecting EBIs projected to be cost-effective but also expected to have the greatest positive health impact among subpopulations experiencing disparities, or alternatively, balancing the ability to achieve CRC screening quality metrics given a limited budget in the short-term. Other themes related to the utility of this tool as an implementation strategy included the following: using the tool to engage health partners in discussions about their EBI implementation options and preferences, reinforcing or strengthening prior recommendations or decisions about EBI selection, and increasing the prioritization of CRC screening compared to other types of preventive healthcare.

### Features of tool that facilitate EBI implementation decision-making

Decision-makers interviewed described the specific features of the simulation tool that aid in the tool’s aforementioned uses for informing EBI implementation (Table 4). The inclusion of both the costs and effectiveness (i.e., health outcomes) of simulated EBIs was identified as a key feature. In particular, interview participants described the importance of including what it will cost to implement each simulated EBI. Respondents also highlighted the reporting of model outputs across multiple time horizons, as well as the breakdown of model outputs by specific subpopulations to target screening gaps and support equity initiatives, as features that support EBI implementation decisions. Additional features promoting evidence-based decision-making included the tool’s visualization of model outputs, focus on population-level health approaches, and ability to “choose your own adventure” (i.e., employ the interactive features or customize use of the model).

**Table 4:** Themes regarding the features of the simulation tool that support EBI implementation (*n* = 17)

Theme	Theme description	# (%) Respondents	Exemplary quote
Inclusion of costs and effectiveness	Importance of reporting both what it costs and the health benefits to be gained with EBI implementation	13 (76%)	“I think it will be useful for people to know which implementation strategies [are] more cost effective and [have] a greater health impact.”
Inclusion of short-term and long-term outcomes	Importance of modeling different time horizons to support decision-making	10 (59%)	“So not just saying…hey, I think this intervention is going to be…the best for our organization and it’s affordable and sustainable, but also being able to give…a projection that matches that, so that way they could see the long-term, not just the short-term.”
Breakdown by subgroups	Utility of being able to review the model outputs for specific subpopulations (e.g., by geographic location or insurance type)	9 (53%)	“…the way that you have broken it up by sex, race, geography is fantastic because a lot of times we are really trying to get clinics to target populations where we’re seeing gaps in screening, or you know the rural is super helpful.”
Visualization of the expected benefits	Visualization of model outputs in charts and graphs	7 (41%)	“…it’s a very nice visual way of showing…these [EBIs] really work and…the actual expense of them is manageable.”
Focus on population health	Opportunity to consider population-level approaches to improving CRC screening and related outcomes	6 (35%)	“…we could utilize this as we’re…talking through like global strategies for addressing CRC screening in underserved populations on a more…statewide level.”
Customization	Utility of being able to adjust the model assumptions	6 (35%)	“I love the choose your own adventure because then partners can go back and say, okay, I have this much or, oh, we want to save this many lives or whatever it is that they’re striving for.”

### Challenges of using tool for informing EBI implementation

Table 5 presents the identified themes regarding obstacles to using the simulation tool to guide EBI implementation. Participants described the use of academic and research language as limiting the tool’s practical use and ability to make the data actionable. Another challenge related to contextual differences between the populations and contexts that were simulated and the decision-makers’ regions and populations served, creating uncertainty over whether the simulated findings would translate to their own service area. Decision-makers reported uncertainty about how to implement the simulated EBIs due to differences in design of those EBIs; for example, they raised questions about which specific characteristics or types of patient reminder programs we simulated in order to replicate those EBIs. Decision-makers also identified the following possible challenges to making decisions using the simulation tool: lack of clarity about the model structure and how outputs were obtained; uncertainty about how to best utilize the option to modify the model assumptions; exclusion of other possible EBIs, especially additional combinations of EBIs, under consideration; limited options for subgroup analysis to support equity goals; and uncertainty about the types of screening modalities accounted for in the model.

**Table 5:** Themes regarding the challenges of using the simulation tool to inform EBI implementation, and recommendations for addressing these challenges (*n* = 17)

Theme	Theme description	# (%) Respondents	Exemplary quote	Recommendations for improvement
Too academic or research focused	Tool needs to be more practical and action-oriented to be used by diverse decision-makers	13 (76%)	“It’s too much about the perfection of the modeling. It’s too statistical and not enough about, okay, here’s what you do with this, like now go forth with everything you need to actually take action on this.”	-Use more practical language (e.g., use lay language instead of terms like “relative risk”) -Provide tutorial/website navigation support -Make data more actionable
Contextual differences across populations and settings	Lack of confidence that EBI implementation will be associated with similar results in the local setting given contextual differences	12 (71%)	“Because I would want to know that the program we’re working on and selecting these best practices would have those similar outcomes. I think you need very similar inputs in order to make a comparison.”	-Provide guidance on model selection and relevancy -Allow users to enter their own model inputs (e.g., local population characteristics) and test impact on model outputs
EBI design differences	Lack of clarity about how each type of EBI was modeled in order to implement it locally	10 (59%)	“Like what are you calling navigation in here? It’s like what’s the recipe, right? Like that’s the chocolate cake, but what ingredients did you put in that chocolate cake?”	-Provide a how-to guide for EBI implementation -Link to other resources that provide guidance on how to implement the selected EBI(s) -Integrate this tool with other implementation strategies
Clarification needed on model components and assumptions	Uncertainty about how the model outputs were obtained and the level of detail that was built into the model structure	9 (53%)	“And then also when we are talking about averting [CRC] cases in the future, what is in that? Are we discussing polyps that we found, the adenomas, the size of them? Like what is kind of going into that?”	-Provide a more simplified overview of how the simulation model works -Clarify how longer-term model outputs (e.g., CRC cases, CRC deaths) were estimated -Call more attention to model assumptions (e.g., assumptions about EBI uptake, adherence to follow-up care, etc.)
Guidance needed on how/why to modify assumptions	Uncertainty about how and when to modify the EBI cost and effectiveness assumptions	8 (47%)	“So how would a user of this tool know to go up or down for the cost multiplier?”	-Walk users through simplified examples of why they would modify the model assumptions and how they would select the adjustments
Not comprehensive of all EBIs	Tool includes a limited menu of individual and combined EBI scenarios	6 (35%)	“Let’s say, for instance, an organization was interested in implementing [a] multi-level strategy that wasn’t in a combination that you prescribed, that could be a functionality that could actually be quite useful down the road.”	-Simulate additional EBIs, such as the use of prompts before mailed outreach or different types of patient reminders (e.g., mail vs. text vs. phone) -Allow users to select combinations of EBIs (i.e., layering on multiple EBIs)
Additional information is needed to support equity	Even more granular detail is needed to prioritize advancing health equity	6 (35%)	“I would like to see more calling out to health equity or really raising the bar in specific areas…so showing how does patient navigation affect like racial impact of health equity for colorectal cancer screenings, because…having [equity] always a part of the conversation, not an add on but weaved within it.”	-Report data at a more granular geographic level (e.g., county-specific outcomes) -Include ability to combine or layer on multiple demographic characteristics (e.g., by race and geography)
Clarification needed on modalities simulated	Tool simulates a limited number of testing modalities	4 (24%)	“And then we should probably…have Cologuard in there, and then future blood tests as well.”	-Simulate the use of modalities other than FIT [fecal immunochemical testing] and colonoscopy -Clarify assumptions about the sensitivity and specificity of different modalities

### Recommendations

For each theme related to possible challenges of using the simulation tool for decision-making purposes, interview participants provided recommendations for how to address these challenges and strengthen the overall utility of the tool (Table 5). These recommendations ranged from minor changes to the tool text to features that can be added to support users to larger structural changes. For example, to make the tool less academic and increase its utility for diverse audiences, decision-makers interviewed identified research-specific terms (e.g., relative risk, life-years) causing confusion and how to rephrase them using more actionable language. In addition, they recommended providing website support, such as tutorials, to navigate decision-makers through using the tool. In terms of contextual differences creating uncertainty about the applicability of model outputs to local settings, participants recommended providing increased guidance on how to select a model and how to assess or have confidence regarding whether they can expect to experience the same outcomes locally. As a more structural change, participants also recommended creating another interactive component where users can enter their own model inputs (e.g., population size, demographics) and determine the expected change in screening and outcomes in their specific context.

## Discussion

This study found that an interactive simulation modeling tool has potential to guide decision-makers across different types of health organizations on EBI implementation decisions. Study participants highlighted the tool’s utility in informing decisions during the early implementation phases, especially around evaluating the evidence base, selecting which individual or multicomponent EBIs to adopt, prioritizing goals for selected EBIs, and making a case for EBI implementation to decision-makers. Participants identified limitations of the tool’s utility in terms of guiding *how* selected EBIs are implemented, but provided recommendations for overcoming some of these challenges.

In their initial reactions to the Cancer Control PopSim tool following the demonstration, participants reported mixed or neutral feelings regarding two areas: (1) comprehension of the simulation tool and (2) whether the tool helps to think differently about strategies for improving CRC screening. Their qualitative responses helped to understand why participants were, on average, neutral about these two areas. In terms of tool comprehension, participants’ feedback on whether others working in the healthcare field could quickly learn how to use the tool depended on which audiences they perceived as the target users. Those who recommended targeting decision-makers working at the higher state level or for large and integrated health systems had more confidence that these individuals would be able to use the tool independently, whereas they felt that healthcare workers in non-leadership roles and/or smaller health organizations may need more assistance navigating this tool due to competing demands and limited experience with the research language used. Regarding their own understanding of the tool, participants identified areas of uncertainty about how the model was developed and its inputs and assumptions. Some of this information was included on the website but not addressed fully during the demonstration. Thus, it is likely that their response may change following their ability to use the tool independently.

With respect to thinking differently about strategies for improving CRC screening, participants’ responses may have been affected by a few different factors. Due to their strong backgrounds in CRC prevention and prior experience implementing or working with health partners to implement EBIs in this area, all participants were familiar with the types of EBIs simulated using this tool (and, therefore, their opinions on how to improve CRC screening may not have changed). Another theme was using the tool to reinforce existing recommendations about which types of EBIs should ideally be implemented. That is, the tool did not change their thoughts about strategies for improving CRC screening, but could be used to make a stronger case to health partners and other leaders about why to implement certain EBIs. Furthermore, participants expressed interest in being able to layer on multiple EBIs that were simulated. Individuals reporting this theme were committed to EBI implementation and felt that it was critical to be able to routinely implement multicomponent or bundled intervention strategies. Since our North Carolina-specific model focused specifically on bundled strategies [19], the full version of the website may help to address this particular challenge. Given these additional insights into participants’ survey responses, we found that conducting interviews was especially important at this stage of the tool development to understand what decision-makers are looking for with this type of implementation strategy.

In these interviews, we built on institutional theory [28], a type of organizational theory, to understand how decision-makers currently select EBIs to implement and the extent to which this type of tool may allow them to engage in more evidence-informed decision-making. Briefly, institutional theory suggests that organizations implement interventions that are consistent with the values, priorities, and norms of other organizations in their network, whether peer health organizations, funders, or other higher-level organizations. We assessed whether use of this tool supported decision-makers in strategizing about how to achieve their local organization’s priorities related to CRC screening or facilitated re-prioritization of their goals through use of a more data-driven approach. Importantly, participants reported multiple ways in which the tool can be utilized to guide EBI implementation. This included selecting EBIs based on the available cost and effectiveness evidence reported on the tool, and obtaining buy-in from others in their network through sharing the model results. In addition, participants described the ability to use the tool to identify organizational goals for CRC screening, such as meeting quality metrics or reaching groups with suboptimal screening rates, and evaluate which EBIs were optimal for achieving those goals.

Notably, there was some mixed feedback related to evidence-based decision-making. For example, some participants reported opportunities to utilize the tool to facilitate discussions with clinics and other health organizations about EBI implementation. In these cases, the decision-makers felt it is important to proceed with whichever EBIs their partners are willing to implement; however, they still found value in working through the tool with their partners as an opportunity to discuss the range of possible EBI options and address questions about the feasibility and impact of implementation. In this way, the tool would support more pragmatic data-driven decision-making. Continued research is needed to evaluate whether tool utilization is associated with EBI adoption or a change in EBI implementation plans.

These interviews also identified opportunities to build on the tool’s strengths for guiding EBI implementation to better support decision-makers’ needs. For example, one theme was the tool’s utility for guiding equity-based decisions about EBI implementation because the model outputs are reported by different demographic and geographic factors. Yet, participants also felt that equity was not sufficiently woven into the tool and identified the lack of even more granular data or the option to evaluate the findings by multiple demographic characteristics simultaneously as a missed opportunity. From this feedback, we learned that the tool can be optimized by expanding its existing focus on disparities in CRC screening and longer-term outcomes to account for additional factors. We can also further clarify on the tool how its interactive portions can be used to support equity goals.

Another primary takeaway for enhancing the tool included determining how to best address decision-makers’ interest in data that supports the actual implementation of EBIs once they have decided to adopt one or more EBIs. That is, in addition to the existing information regarding how much it will cost to implement each EBI and descriptions of the types of EBIs simulated, decision-makers wanted to see the “recipe” for exactly how to conduct the implementation activities involved in each EBI. This might include, at minimum, providing more specificity about the particular studies that informed each simulated EBI scenario. As another possibility, participants recommended connecting tool users to existing websites and resources describing the implementation activities [29], perhaps as a final step in our step-by-step process for navigating the tool. Their feedback also supported the potential for integrating this simulation tool with other types of systems science tools, such as process flow diagrams, that can be used to plan for implementation and sustainment in greater detail [14, 15, 30]. Additional research should evaluate how to best integrate these types of tools for maximum benefit related to data-driven decision-making, implementation readiness, and implementation outcomes.

This study included limitations. This was a formative study intended to understand how an interactive simulation tool could be optimized to address the needs and preferences of individuals making decisions about how to improve CRC screening in different contexts. While participants provided insight into how this tool could be used as an implementation strategy, particularly for selecting EBIs, we were unable to test the effectiveness of using this tool on decision-making or implementation outcomes. Future work should test the extent to which use of this tool has an effect on outcomes such as intent to implement EBIs, confidence or satisfaction with decision-making, perceptions about which EBI(s) is preferred, and proportion of the target population screened. Additionally, participants were only able to view a subset of the tool options and types of information reported. Although this more focused approach served to minimize the length of the demonstration and concentrate on the tool’s broader utility, it may have limited participants’ perceptions of how the tool could inform EBI implementation. It is also possible that some of the participants’ questions or recommendations could have been addressed by having increased access to the full tool contents. Our inclusion of multiple types of decision-makers with diverse backgrounds from a mix of organizations may have diluted our understanding of the perspectives of each stakeholder type. Finally, participants reported extensive experience with implementing EBIs. Further research is needed to assess how those who have not yet initiated CRC screening EBIs might perceive or use this tool differently.

In summary, diverse decision-makers reported that the Cancer Control PopSim tool, or related types of simulation tools, can be used to inform CRC screening EBI implementation—particularly in terms of using simulated data to select EBIs during implementation planning. Participants identified a wide range of opportunities to optimize the tool’s utility to support decision-making, including simpler changes like providing additional text information about the model itself and the simulated EBIs to structural changes such as building in additional interactive components. Most importantly, their recommended changes emphasized the need for a more structured approach to actual implementation of the selected EBI and the ability to further tailor the tool to their local contexts.

### Supplementary Information

Below is the link to the electronic supplementary material.

Supplementary file1 (DOCX 22 KB)

## Data Availability

The datasets analyzed during the current study are available from the corresponding author on reasonable request.
