# Supplementary material for: Engaging stakeholders in the use of an interactive simulation tool to support decision-making about the implementation of colorectal cancer screening interventions
Source: Cancer Causes Control. 2023 May 6;34(Suppl 1):135–48. doi: 10.1007/s10552-023-01692-0 (PMC10689514; doi:10.1007/s10552-023-01692-0)
Supplement: Supplementary file 1 — Supplementary file1 (DOCX 22 KB) [file 10552_2023_1692_MOESM1_ESM.docx]

**Supplemental File: Semi-Structured Interview Guide for Interactive Simulation Tool**

Introduction

Thank you for agreeing to participate today. In this study, we are interested in learning what you and other participants think about an interactive, web-based simulation modeling tool and the impact it could have in informing the selection and implementation of strategies for improving colorectal cancer (CRC) screening. By simulation modeling, we mean a computer model that uses data to create a virtual representation of reality and to forecast or project future outcomes.

Participants Characteristics

1. What is your current occupation as it relates to CRC screening?
   1. How long have you been in this role?
   2. What is your role related to decision-making about CRC screening programs at your organization?
2. How, if at all, have you or your organization previously used or considered cost-effectiveness analyses or simulation modeling results when making decisions about strategies for improving CRC screening?

Tool Demonstration

I am now going to share with you a short demonstration of a newly developed web-based simulation tool, called Cancer Control PopSim. Afterwards, I will ask you some questions about how you might use this tool when making decisions about strategies for improving CRC screening. As I walk through the demo, if you have any clarifying questions, please go ahead and ask them. You will also have an opportunity to ask more detailed questions later.

The full Cancer Control PopSim website, which you should see on your screen now, includes background information about our modeling approach, prior research, and our workgroup. Today, we will focus on the interactive portion of the website only, located here under “Try it”.

Please note that following today’s demo and interview, you will have an opportunity to use this tool on your own before completing a follow-up survey. For now, I will give a high-level orientation to the types of information included and the interactive features available.

Getting started, this tool provides an opportunity to understand and compare the expected impact of implementing strategies for improving CRC screening and outcomes. Through simulation modeling, we projected the short-term and long-term impact of these strategies if implemented in different populations and settings. We will focus on implementing these strategies in a population of Oregon Medicaid enrollees. Once live, this tool will also report the results if implemented among the full North Carolina population.

This table provides an overview of our Oregon Medicaid model. The population includes approximately 68,000 Oregon residents enrolled in Medicaid accountable care organizations, aged 50 to 64 years, and not up-to-date on CRC screening. We assumed that strategies would be implemented over a 5-year period. Our goal was to understand, using simulation modeling, what would happen in this population in terms of screening and health outcomes in the future if various strategies were implemented.

Here, we can see the strategies that were simulated. For each strategy, you can expand the box to see what was included and what we assumed in terms of how effective the strategy will be, compared to doing nothing, and how much it will cost to implement. You can also see a breakdown of the specific cost components. This link provides additional information on the specific sources used to derive these estimates.

Next, we can select the specific strategies that we want to see the results for. While all these strategies will be available once the tool is live, for now, we included a few examples for testing purposes. I’ll go ahead and select all three strategies: sending patients reminders, navigating patients to overcome barriers to screening, and the combination of sending stool kits to patients’ homes plus navigation. As we go through the tool, we will compare each of these strategies to usual care, in other words, the absence of any intervention.

As a last step before seeing the results, we can explore the specific assumptions that were made about the effectiveness and the cost of each strategy. In our simulation modeling studies that informed this tool, we made assumptions about each strategy’s effectiveness and cost based on prior studies conducted in similar populations. However, you may wish to scale up or down these estimates for various reasons, for example if you have reason to believe a particular strategy would work better in your setting or perhaps cost less, due to having some existing infrastructure already in place.

Starting with how effective each strategy will be, the top row of this table summarizes what we assumed in terms of the relative risk for each strategy. If you decide to adjust the relative risk by, say, +0.1, the relative risk for patient reminders, as an example, will increase from our assumption of 1.26 to 1.36, thus scaling up the effectiveness. This would mean that individuals are 36% more likely to be screened with this strategy compared to usual care. To make these adjustments, you can use these drop-down menus. For now, I will stick with the default setting – what our team assumed – for each strategy.

Similarly, you can scale up or down the cost to implement each strategy. Again, this top row presents what we assumed about the costs, noted with a cost multiplier of 1. If we were select a multiplier of 1.5, for example, the implementation cost for patient reminders will increase from $1.43 to $2.15 per patient. As before, you can use these drop-down menus to change the multiplier, but we will go ahead with our default setting.

Finally, we can see the results. Starting with the change in CRC screening, we can view the percentage of the population up-to-date on screening under usual care, and the expected change for each selected strategy. We can also look at the change in screening for specific groups – by sex, by race, by ethnicity, by geography (urban vs. rural), and by age. Note that, if we had decided to adjust our model assumptions, these results would be updated to reflect our changed assumptions. And this will be the case throughout the tool.

Next, we can see longer-term health impact. Here is the number of CRC cases expected over these individuals’ lifetimes under usual care. And here is the number of cancer cases averted for each strategy.

Similarly, we can look at the total life years expected in this population under usual care, and the number of life years gained for each strategy. Life-years are the number of remaining years of life across all individuals in this population based on each individual’s life expectancy. As before, we can see these results for specific groups. We can also look at the breakdown by cancer stage at diagnosis.

Next, we can look at costs. Here are the costs of each strategy over the 5-year period. The navy blue shows the costs required to implement each strategy. The bright blue shows the costs associated with the actual screening tests and any follow-up required. And, the green shows the costs of cancer treatment. Below the charts, you can also see the total costs, including each of these three categories.

Then, we can see the cumulative costs by year in these graphs. As you can see, the costs are typically decreasing over time, compared to usual care. We will be expanding this timeframe to look at the costs over these individuals’ lifetimes, but for now are showing the data in a 5-year period.

And, finally, we can ow estimate the cost-effectiveness of each strategy compared to usual care. Once live, users will have additional options to select from these menus. For now, we are going to focus on the cost per additional person-year up-to-date on CRC screening during a 5-year period. Each data point in this chart represents the incremental cost-effectiveness ratio (or the ICER) for an intervention compared to usual care. For example, it will cost $30 in order to gain one additional person-year up-to-date on screening for patient reminders, compared to usual care. The data points that fall on or below this line are those that are considered cost-effective, but this will depend on what decision-makers are willing to spend per health outcome gained.

Thank you for viewing this demo. Do you have any initial questions before we proceed with the interview?

User Testing

1. We are interested in how people might use tools like this for decision-making about strategies for improving CRC screening. This next set of questions will focus on your initial reactions about the utility of this tool. Please rate each of the following from 1 (Strongly Disagree) to 5 (Strongly Agree).

| 1 | 2 | 3 | 4 | 5 |
| --- | --- | --- | --- | --- |
| Strongly disagree | Disagree | Neither agree or disagree | Agree | Strongly agree |

1. I think that I would like to use tools like this to support decision-making about strategies for improving CRC screening frequently.
2. This tool makes me think differently about strategies for improving CRC screening.
3. I found the various components in this tool were well integrated.
4. I think that I would need to learn more about simulation modeling to be able to use this tool.
5. I would imagine that most people in healthcare could learn to use this tool quickly.
6. I think this tool could help to implement strategies for improving CRC screening more effectively.

Perceived utility and challenges of using tool as an implementation strategy

1. What do you think are the primary benefits of a simulation tool like this one?
   1. Which individuals or organizations do you think would find this tool most useful? Why?
   2. How might you use this tool when making decisions about strategies for improving CRC screening?
2. What do you think are the primary challenges of a simulation tool like this one?
   1. What might prevent you from using this tool to support decision-making about strategies for improving CRC screening?
   2. What other kind of information or support would you need to use this tool?
3. Considering your other strategies for informing efforts to improve CRC screening, how might you use this tool in your decision-making role?
   1. How do you typically approach decision-making? How would this complement or substitute your usual approach?
   2. Ideally, how would you envision being able to use this tool in your own context?

Visual appearance

1. What did you like about the presentation (i.e., visual appearance) of the simulation results? Why?
2. What did you dislike? Why?

Comprehension of results

1. After this initial demonstration of the tool, how easy or difficult did you find it to understand the model results? What would help to make it easier to understand or interpret?
2. Do you think there are any other important factors affecting outcomes associated with these evidence-based practices that are not already accounted for in the model? If so, what is missing?

Comprehension of interactive tool instructions

1. How easy or difficult did you find it to understand the instructions about how to modify the model assumptions? How could the instructions be made easier to understand or interpret?

Additional suggestions

1. Are there any resources or external tools (e.g., websites, trainings, etc.) that you would suggest including on our site? Which ones and why?
2. Do you have any other recommendations for how we can better support decision-makers like you in using this tool to guide implementation of strategies or programs for improving CRC screening?
3. Is there anything else you would like to share about this tool?
